# Analyses of Vibration Signals Generated in W. Nr. 1.0038 Steel during Abrasive Water Jet Cutting Aimed to Process Control

**DOI:** 10.3390/ma15010345

**Published:** 2022-01-04

**Authors:** Martin Tyč, Irena M. Hlaváčová, Pavel Barták

**Affiliations:** 1 Department of Physics, Faculty of Electrical Engineering and Computer Science, VSB-Technical University of Ostrava, 17. Listopadu 2172/15, 70800 Ostrava-Poruba, Czech Republic; 2 Kámen Ostroměř, Nádražní 414, 50752 Ostroměř, Czech Republic; bartak@piskovce.cz

**Keywords:** abrasive water jet, vibration signals, AWJ machining, process control

## Abstract

The presented research was aimed at finding a suitable tool and procedure for monitoring undercuts or other problems such as cutting without abrasive or inappropriate parameters of the jet during the abrasive water jet (AWJ) cutting of hard-machined materials. Plates of structural steel RSt 37-2 of different thickness were cut through by AWJ with such traverse speeds that cuts of various qualities were obtained. Vibrations of the workpiece were monitored by three accelerometers mounted on the workpiece by a special block that was designed for this purpose. After detecting and recording vibration signals through the National Instruments (NI) program Signal Express, we processed this data by means of the LabVIEW Sound and Vibration Toolkit. Statistical evaluation of data was performed, and RMS was identified as the parameter most suitable for online vibration monitoring. We focus on the analysis of the relationship between the RMS and traverse speed.

## 1. Introduction

Abrasive water jet (AWJ) is one of the progressive technologies which has spread to many industries during the last several decades. The antecedent of AWJ was groundbreaking research by Franz in high pressure water jet cutting lumber in the 1950s, resulting in pure waterjet invention [1]. In the end of the 1970s, Hashish started adding abrasiveness to the jet and thus achieved high efficiency so that in the early 80s, it was possible to start cutting steel, concrete, etc. [2]. The main advantages of AWJ are the insignificant thermal influence of most materials [3], extremely low increase in the internal stress of the material [4], and the versatility of the jet as a tool capable of cutting almost any material [5]. Today, AWJ machining technology is high tech due to intensive developments in many companies. However, due to the expanding market and increasing demand for special cutting by AWJ such as 3D machining and machining of hard-machine materials [6], it is necessary to develop a reliable procedure for monitoring the cutting process.

Many authors were studying this topic, and different, more or less effective methods were proposed. Jurisevic et al. [7] measured the emitted sound generated during the AWJ straight cut operation on aluminum alloy plates of two different thickness and analysed its characteristic attributes. Axinte and Kong [8] applied a very complex method based on evaluation of signals detected by an array of energy-related sensors, namely acoustic emissions (AE); three AE sensors were mounted along the jet trajectory (nozzle, workpiece fixture and dummy plate below the workpiece), and their measurement was amended by two dynamometers, mounted under the workpiece and dummy plate, measuring the impingement of the direct and idle jet, respectively. The whole system was aimed to detect various types of malfunction of the device, i.e., nozzle clogging, non-uniform jet penetration during through cutting operations and nonconstant jet eroded footprint during milling operations. Krenický and Rimár [9] measured vibrations on several parts of the machine, trying to find relation to striations formed at the cut surface. Hreha et al. [10] measured vibrations on the workpiece and, applying polynomial multi-parametric regression, prepared a formula for calculation of surface roughness from the traverse rate of the cutting head and average of absolute values of acceleration amplitudes. Similar research was realized by Sutowski et al. [11]. Mikler monitored undercut using acoustic emissions [12] and questioned reliability of direct connection between AE and surface quality. Recently, Pahuja and Ramulu [13] analysed the AE signals using wavelet packet transform (WPT) and proposed an algorithm to identify and characterize these signals. Copertaro et al. [14] aimed at demonstrating that vibroacoustic sensors are the right choice for monitoring the AJW cutting capability; however, the investigation has been carried out with the cutting head in a steady position, while its movement was found to negatively affect the performance of the method. Therefore, it seems to serve rather as a calibration method.

Thus, in spite of great effort of many researchers, so far, commercially manufactured monitoring equipment has not been launched on the market. For work in the industry as well as in laboratories studying AWJ, it would be a great benefit to have technology capable of online monitoring of the depth of cut of the work-piece. The abrasive water jet is able to cut thick materials such as steel or rock, but for very thick or hard-machine materials, it can be difficult to correctly determine the parameters of the jet. Then it is important to set the parameters of AWJ for an economically tolerable cut. Separating cut (without the need for cutting quality) is widely used, and it is necessary to cut the shortest time as possible. Online monitoring of the cut should provide clear answers such as the instantaneous depth of the jet during drilling or if, during cutting, undercut occurs, or whether the jet cuts through the material unnecessarily long and uneconomically.

During the cutting of materials with an abrasive water jet, a water jet is used as the medium which carries the abrasive. The abrasive is a component of the jet that erodes the material. The abrasive particles fall on the surface of the material, where the material is removed by erosion. The kinetic energy of these particles is partly used to break the bonds of the material; the part is transferred to the surroundings in the form of acoustic oscillations; part of the energy is converted into heat, plastic deformation of material, etc. When the machine has incorrectly set parameters, the material is not cut properly, less energy is consumed to breaking bonds, and more energy remains in material. The energy should, therefore, spread through the material in the form of AE and can serve as a source of information about type of mechanical wear mechanism passing in the material [15], or could lead to increased vibration of the workpiece. These vibrations can be measured using accelerometers and help to detect malfunction of the system such as undercuts, lack of abrasive or pressure drop. Although acoustic signal characteristics exhibit stronger relationship with the process characteristics, the implementation is limited by the location of the respective sensor on the part [16]. Moreover, depending on the nature of the source event, various percentages of the total energy are available as measurable acoustic waves [17], so the evaluation of the measured data is too complex to be applied as a quick, simple and economic diagnostic tool.

We categorise the measuring techniques for the monitoring of machining operations into two approaches: direct and indirect. In the direct approach, the actual quantity of the variable, most often the tool wear, is measured. However, due to the practical limitations caused by access problems during machining, illumination and the use of cutting fluid the direct methods can often be used only as laboratory techniques [18]. The indirect measurements often use various types of sensors to determine auxiliary quantity which enables us to deduce values of the actual quantity through empirically determined correlation [19]. Accelerometers represent one of the most often used type of sensors used for detection of vibration during machining operations. The main advantage of the accelerometer is its linearity over a wide range of frequencies, which enables us to identify either the condition of cutting operation [19,20] or monitor the cutting tool condition [21] and prevent its sudden breakage [22] or malfunction [23,24]. Accelerometers are widely used also in other technical applications [25]; therefore, one can utilise a wide spectrum of knowledge concerning signal processing and interpretation.

In this research paper, we deal with the measurement of vibration using piezoelectric accelerometers fastened to the workpiece. These vibrations should help for monitoring the cut of hard-machine material and thus save on abrasive and time when working in the laboratory. We performed the experiments in the laboratory of AWJ of the Department of Physics VŠB-TUO. A measuring system from National Instruments (NI) and piezoelectric accelerometers from PCB Piezotronics were used while searching the relationship between vibrations and cuts. Our goal was to find a simple, low-cost and time-saving method of malfunction detection usable in practice, similar to that in the research work of Postel et al. [26]. Based on the results, we plan to design a system for online monitoring of cuts, during cutting thick or hard-machine materials. We believe that an important part of optimizing the drilling and cutting of hard-machine materials is to be aware of the relationship between the interaction of the jet with the material and the observed vibroacoustic emissions and to identify their manifestations in the measured signals.

## 2. Materials and Methods

The technical equipment of the AWJ laboratory of the Department of Physics VŠB Technical University of Ostrava consists of the abrasive water jet PTV WJ1020-1Z-EKO and the high-pressure pump PTV 19/60; with pressure up to 415 MPa. An NI measuring system was used to record the signals from the accelerometers. The measurement was realized on an assembly consisting of a five-slot NI PXIe-1073, a NI PXIe-4492 data acquisition module, and an NI PXI-Express Card 8360, which was used to connect a PC to an A/D converter. We use NI Signal Express software for data recording, and LabView Sound and Vibration Assistant for online signal analysis and recording.

**Figure 1:**
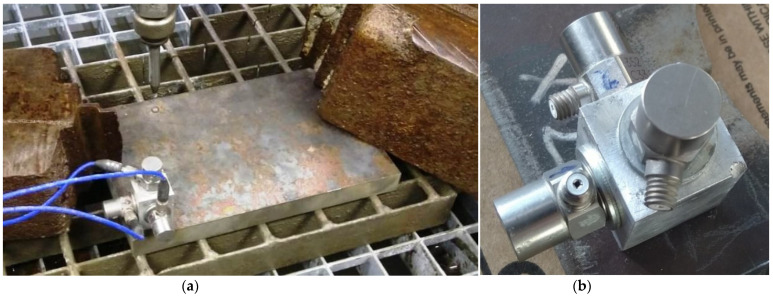
(**a**) fixation of the measuring block to the cut material; (**b**) detail of the measuring block.

We measured vibrations using three PCB 352C33 accelerometers. To connect the accelerometers to the workpiece, we made a special block where the accelerometers are arranged in three mutually perpendicular axes [X, Y, Z]. This block is fastened to the workpiece with a single screw (Figure 1). The advantage of the block lies in the simplicity and speed of mounting the three accelerometers on the workpiece and the possibility to keep the mounting of the accelerometers in the desired position even if the body shape is irregular. In an upgraded version, the block was equipped by a special magnet for attaching accelerometers block. An additional upgrade was designed to simplify accelerometers mounting to ferromagnetic materials, making measurement more flexible. Its benefit is a nondestructive way of attachment.

We used abrasive Australian garnet mesh 80, water pressure was 380 MPa, nozzle diameter 0.25 mm, focusing tube length and diameter 76 mm, and 0.76 mm, respectively. The standoff distance was 2.5 mm (Table 1). The signal sampling frequency was 30 kHz.

The material used for the research was structural steel W. Nr. 1.0038 (DIN RSt 37-2) (Table 2) on plates with 5 different thicknesses. This material is classified as non-alloy low carbon steel and it is widely used as a construction material thanks to its relatively low price and easy availability [27].

**Figure 2:**
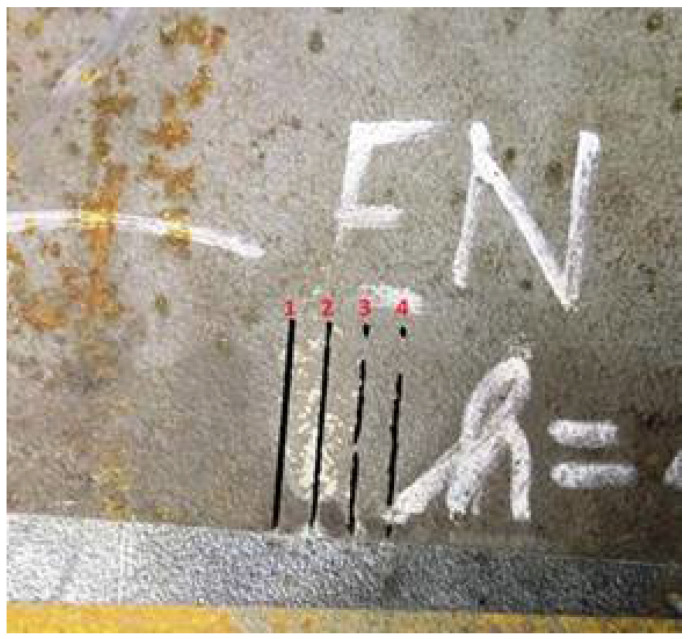
Example of cuts performed with increasing traverse rate and therefore deteriorating quality: the first one from the left represents excellent cut; the second one good cut; the last two indicate poor quality, containing undercuts or bridges.

The measurement was realized in the original version of accelerometers mounting, i.e., using a measuring block fixed with a screw. The dimensions of the cut samples were 109 mm × 168 mm, the block with accelerometers was attached in the corner of the workpiece. The AWJ machine cut the material in the X direction, the Y direction was the second lateral accelerometer, Z was the axial (vertical) accelerometer, i.e., parallel with the jet axis. We always measured four sections, 25 mm long, the cuts were performed with four different speeds (Table 3). The speeds were increasing, they were designed to produce kerfs with degrading quality of the cut (Figure 2). The measurement cycle was repeated on the same material for all measured thicknesses, the respective traverse rates were recalculated for each thickness according to the Hlaváč’s model, recently updated in [28]. Based on this model classification, the data were divided into four cutting qualities (Table 3).

The spectral power density method often appears in articles referring to monitoring abrasive water jet. Using this method of signal analysis seems logical because it brings us information about the performance of individual frequencies. However, the analysis of such a spectrum brings a lot pitfalls. For online monitoring, it is necessary to create an elementary tool so that the machine operator can simply know if the material is completely cut. Results of signal analysis using spectral power densities are highly variable. Disturbing elements of the surroundings, such as a lathe motor or a cooling fan motor, often appear in the signals. This method is therefore suitable for signal analysis, where we are able to guarantee the same conditions for all sections, and the resulting analysis is performed after the measurement is completed. It proved not to be beneficial for online analysis in the presented research.

**Figure 3:**
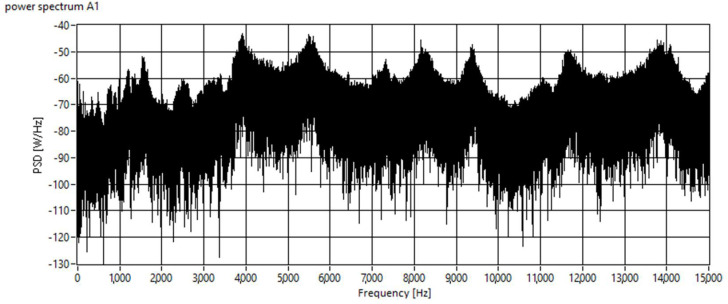
Typical power spectrum of material, it is difficult to identify individual sources of interfering idle signals.

At the beginning of the research, a program for signal analysis using spectral density was prepared, and several signals were processed to verify its functionality. It came out that the spectra did not change according to some features typical of the section. Figure 3 shows a typical power spectrum of the material.

Therefore, RMS time signal was chosen for further processing, due to simpler and clearer analysis, which is very convenient for online analysis. The vibrations measured by the accelerometers were evaluated in the LabView software, and root mean square (RMS) of the assigned section was calculated. The RMS is defined in Equation (1).

(1) $$y_{ef}=\sqrt{\frac{1}{T}\int_{0}^{T}y^{2}}dt$$

The RMS value is a measure of the energy transmitted by a signal. It can only acquire positive, nonzero and finite values. In our previous research [29] signals were analyzed by FFT, but it came out that this form of analysis should be too complicated for industrial use. On the other hand, the background of the measured signals in our laboratory appeared to be constant and does not seem to affect the use of the time signal and RMS. Therefore, for the best approximation to cutting in practice, the same conditions were used as in industry and the measurement was performed without further adjusting conditions.

Four different cuts with traverse rates corresponding to four required cutting qualities (excellent, good, separating and limit) were performed for each thickness of material, which represents 20 measurements of time signals used for RMS evaluation. Preliminary tests did not indicate any substantial change in the RMS value for the repeated cuts under the same operating conditions; therefore, a single run-cut for each combination of traverse rate and material thickness was used for evaluation.

## 3. Results and Discussion

**Figure 4:**
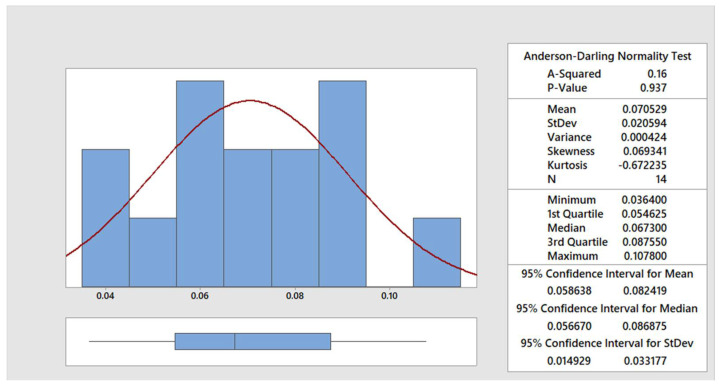
Summary report for excellent quality.

**Figure 5:**
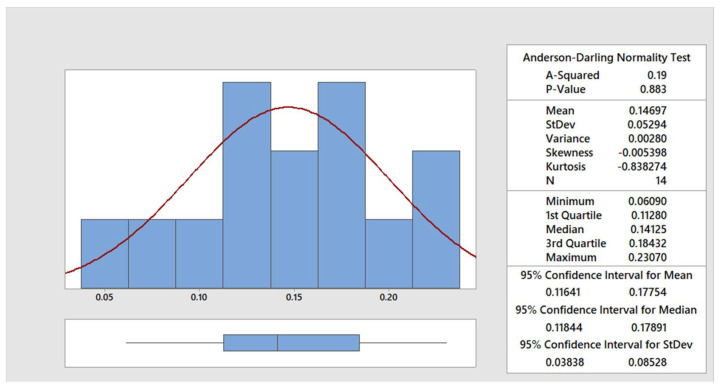
Summary report for good quality.

**Figure 6:**
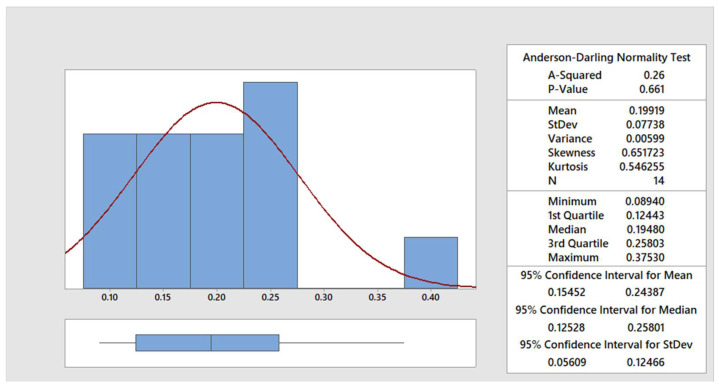
Summary report for separating cut.

**Figure 7:**
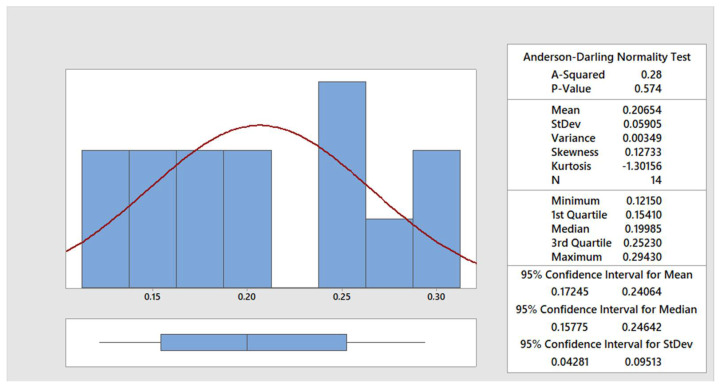
Summary report for limit cut.

The data were statistically processed using the Anderson–Darling normality test [30]. In the Figure 4, Figure 5, Figure 6 and Figure 7, the graphical summary of the statistics for each category of cut quality is presented, namely the normal distribution curve and histogram. A table of basic statistical processing results is included in each picture, indicating that the data come from a normal distribution.

The variances are different and the normality was not rejected, so we proceeded to the exact test of homoskedasticity—the Bartlett test [31]. At the significance level of 0.05, we reject the assumption of agreement of sector deviations. There is a statistically significant difference between the variances of the individual groups of sections. The conditions are suitable for and the Kruskal–Wallis test [32] (Table 4). The results will be discussed in the following section.

**Figure 8:**
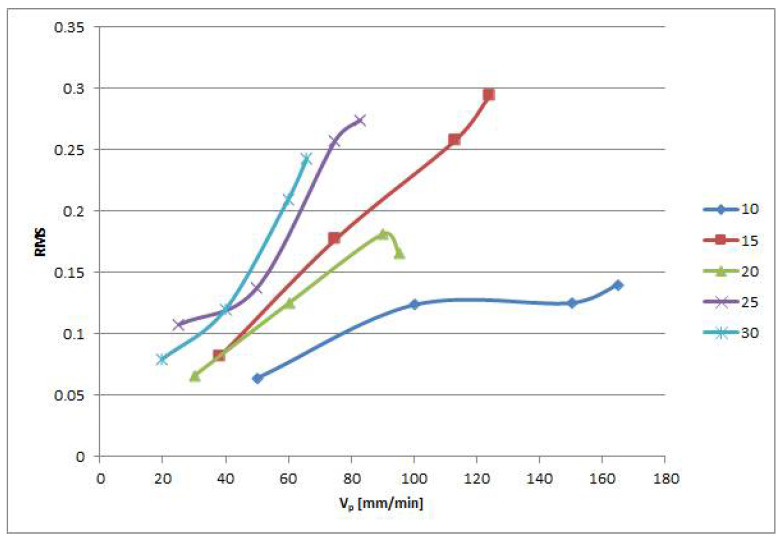
RMS change with traverse speed for different thicknesses of steel.

The results of the above-mentioned statistical tests proved that the RMS changes statistically significantly with the traverse speed; it can be related with the quality of the cut. Figure 8 presents a graph of the RMS dependence on the traverse speed. Each material thickness has its own curve. We can see that the general trend is that RMS increases with the growing traverse speed. There are two results which deviate from this trend. Excellent cutting quality results for 25 mm thickness seem to be inhomogeneous in RMS compared with other cutting qualities. This phenomenon might arise from the step increase of the RMS after over-running of the optimal cutting speed, while the growth trend can be slower at higher speeds. In the 20 mm thickness curve, we can see that the RMS dropped down for the fastest cut. However, this exception might appear due to inhomogeneities in the material or another similar unexpected event that caused it. We should note that the separating speed is only 10% lower than the limit one.

**Figure 9:**
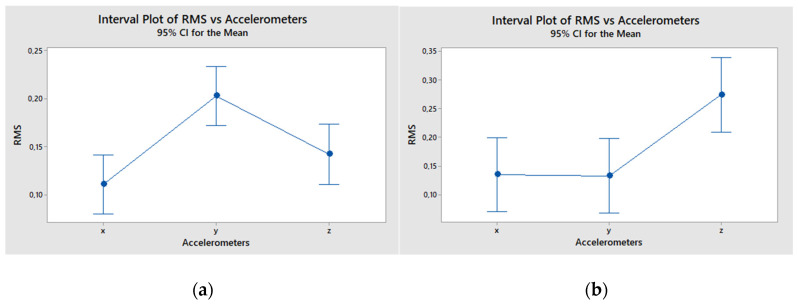
(**a**) Analysis of variance of RMS sorted through axes-measurement in Ostrava; (**b**) Analysis of variance of RMS sorted through axes-measurement in Milano; the cutting on duralumin 25 mm thick, with variable values of pressure and abrasive mass flow rate.

Furthermore, we wanted to find out, following a thorough statistical analysis of individual directions, whether the measurement of vibrations on the workpiece is dependent on the axis [X, Y, Z], from which we measure vibrations. The RMS values were sorted through the directions (Table 5). After excluding the only outlier (value 0.451108 in the Z column), the analysis of variance (ANOVA) was performed, and statistically significant difference between individual directions was proved. The results based on the Tukey’s honestly significant test [33] are presented in Figure 9a.

In terms of RMS level, it was proved that it depends on location of the accelerometers. Surprisingly, in our laboratory in Ostrava, we found that it was statistically the best to measure the RMS level with the accelerometer Y (normal to surface parallel to the cutting surface) and the worst with the accelerometer X (cutting direction). This might correlate with gradual deterioration of the cut quality and increase in lateral deviations of the jet during close-to-limit cuts, but it should also signal a machine fault. The latter explanation raised from the fact that we have found different results in another measurement scheme realized with another material on cutting machine in Politecnico di Milano (Figure 9b).

Nevertheless, we suppose that for more complex cut shapes, it should definitely be more advantageous to measure with more accelerometers to identify unwilling changes in the cutting process (These changes should then be located directly from the detailed analysis of the time signals.). However, for linear cuts, it is sufficient to have one accelerometer mounted on the workpiece surface, i.e., in the jet axis.

## 4. Conclusions

The statistical analysis of the experimental data confirmed that the relationship of the RMS to the traverse speed is statistically significant. The general trend is that RMS increases with the growing traverse speed; however, inhomogeneities in material might lead to unexpected improvement in cutting and therefore sudden drop down of the RMS signal for high traverse rate.

In the search of the most advantageous accelerometer for measuring vibrations on the machined material, we found that in terms of the RMS level, it depends on the direction in which the accelerometer measures vibrations; it was statistically significantly proved that it was best to measure with the accelerometer Y and the worst with the accelerometer X (which is against the direction of the cut). This finding, however, cannot be generalized; it should be a specific feature of the machine applied in this research.

For industrial use, we recommend to measure with one Z accelerometer mounted on the material surface so that the accelerometer detects vibrations parallel to the jet.

The planned application of the presented conclusions is as follows: in case of unknown thick materials, it should be difficult to choose optimal traverse speed to realize through cutting without undercuts. Additional cutting in the case of several noncut points should be very time consuming and unnecessarily expensive. Continuous monitoring of the RMS signal using a special block with three accelerometers together with evaluation of the time records of the original signals enables us to identify problems and locate them. Additional cutting should therefore be precisely targeted and much more economical.

## Figures and Tables

**Table 1:** Parameters and factors applied in the AWJ cutting.

Variable [Unit]	Value
Pump pressure [MPa]	380
Nozzle orifice diameter [mm]	0.25
Mixing tube diameter [mm]	0.76
Mixing tube length [mm]	76
Abrasive mass flow rate [g/min]	250
Abrasive type	Australian garnet #80
Standoff distance [mm]	2.5
Cutting speed [mm/min]	20–165

**Table 2:** Chemical composition of examined steel in wt. %.

	C %	Si %	Mn %	P %	S %	N %	Cu %
W. Nr. 1.0038	max. 0.19	-	max. 1.50	max. 0.045	max. 0.045	max. 0.014	max. 0.60

**Table 3:** Traverse rates used for different thicknesses and quality of cutting.

Cutting Quality:	Thickness in mm	10	15	20	25	30
Excellent	Traverse speed v_p_	50	38	30	25	20
Good		100	75	60	50	40
Separating cut		150	113	90	75	60
Limit cut		165	124	95	83	66

**Table 4:** The results of the Kruskal–Wallis test.

Cut Quality	N	Median	Ave Rank	Z
Excellent	14	0.0673	9	−5.17
Good	14	0.14125	27.7	−0.21
Limit cut	14	0.19985	39.9	3.03
Separating cut	14	0.1948	37.4	2.35
Overall	56		28.5	
H = 31.05; DF = 3; P « 0.001				

**Table 5:** RMS of individual cuts in Ostrava sorted through direction of measurement.

Thickness [mm]	X	Y	Z	v_p_ [mm/min]
10	0.063765	0.090058	0.063130	50
10	0.123962	0.222298	0.169768	100
10	0.125512	0.23860	0.167805	150
10	0.140289	0.244911	0.184055	165
15	0.068251	0.081516	0.086712	38
15	0.144684	0.177507	0.204797	75
15	0.208192	0.257962	0.451108	113
15	0.195216	0.294343	0.237677	124
20	0.066276	0.112672	0.094817	30
20	0.125219	0.230684	0.177159	60
20	0.181439	0.375277	0.269824	90
20	0.165931	0.293618	0.204544	95
25	0.046918	0.107784	0.057235	25
25	0.060920	0.137836	0.091477	50
25	0.121212	0.258099	0.174038	75
25	0.121463	0.274494	0.158659	83
30	0.036423	0.079713	0.044846	20
30	0.045271	0.119909	0.071335	40
30	0.089390	0.209744	0.111748	60
30	0.088969	0.243477	0.132883	66

## Data Availability

Not applicable.
